# Anti-miR delivery strategies to bypass the blood-brain barrier in glioblastoma therapy

**DOI:** 10.18632/oncotarget.8837

**Published:** 2016-04-19

**Authors:** Dong Geon Kim, Kang Ho Kim, Yun Jee Seo, Heekyoung Yang, Eric G. Marcusson, Eunju Son, Kyoungmin Lee, Jason K. Sa, Hye Won Lee, Do-Hyun Nam

**Affiliations:** ^1^ Department of Health Sciences and Technology, SAIHST, Sungkyunkwan University, Seoul, Korea; ^2^ Institute for Refractory Cancer Research, Samsung Medical Center, Seoul, Korea; ^3^ Department of Neurosurgery, Samsung Medical Center, Sungkyunkwan University School of Medicine, Seoul, Korea; ^4^ Department of Anatomy and Cell Biology, Sungkyunkwan University School of Medicine, Seoul, Korea; ^5^ Department of Urology, Samsung Medical Center, Sungkyunkwan University, School of Medicine, Seoul, Korea; ^6^ Providence Therapeutics, Calgary, Canada

**Keywords:** anti-miR, glioblastoma, intratumoral injection, intraventricular injection, delivery efficiency

## Abstract

Small non-coding RNAs called miRNAs are key regulators in various biological processes, including tumor initiation, propagation, and metastasis in glioblastoma as well as other cancers. Recent studies have shown the potential for oncogenic miRNAs as therapeutic targets in glioblastoma. However, the application of antisense oligomers, or anti-miRs, to the brain is limited due to the blood-brain barrier (BBB), when administered in the traditional systemic manner. To induce a therapeutic effect in glioblastoma, anti-miR therapy requires a robust and effective delivery system to overcome this obstacle. To bypass the BBB, different delivery administration methods for anti-miRs were evaluated. Stereotaxic surgery was performed to administer anti-Let-7 through intratumoral (ITu), intrathecal (ITh), and intraventricular (ICV) routes, and each method's efficacy was determined by changes in the expression of anti-Let-7 target genes as well as by immunohistochemical analysis. ITu administration of anti-miRs led to a high rate of anti-miR delivery to tumors in the brain by both bolus and continuous administration. In addition, ICV administration, compared with ITu administration, showed a greater distribution of the miR across entire brain tissues. This study suggests that local administration methods are a promising strategy for anti-miR treatment and may overcome current limitations in the treatment of glioblastoma in preclinical animal models.

## INTRODUCTION

Glioblastoma is one of the most advanced adult brain tumors and is classified as a World Health Organization (WHO) grade IV astrocytoma. Malignant glioblastoma patients only survive on average less than 2 years from the time of diagnosis [1]. Presently, surgical resection followed by radiation and temozolomide therapy is the only therapeutic option available for glioblastoma patients [2]. However, tumors invariably relapse, and the current standard treatments have limited options when such events occur [3]. Although several targeted therapeutics are currently under preclinical and clinical development, the delivery method must address the blood-brain barrier (BBB), which makes systemic treatment more difficult [4]. Because of this, there is still an unmet need for the development of a novel treatment strategy for glioblastoma patients.

miRNAs are small noncoding RNA molecules that consist of 19–24 nucleotides. Usually, miRNAs bind to their target mRNA in the 3′ UTR via a post-transcriptional regulation process that results in decreased expression of the target mRNA [5]. Recent reports have highlighted the roles of miRNAs in the regulation of various biological processes including cancer cell proliferation, invasion, metastasis, and stemness in different cancers including glioblastoma [6–9]. miRNAs have therefore emerged as a class of promising therapeutic targets for cancer treatments [10–12]. Anti-miR treatment, however, cannot cross the BBB, limiting its use when applied via the traditional systemic approach [13, 14]. Essentially, the development of anti-miRs has remained a challenge, since they cannot be delivered to the brain. To maximize the therapeutic benefits of oncogenic miRNA inhibition, different methods need to be developed for glioblastoma patients [15, 16].

To evaluate methods of anti-miR administration in glioblastoma xenograft models, the anti-Let-7 oligomer was employed in this study. The Let-7 family (Let-7a, Let-7b, Let-7c, Let-7d, Let-7e, Let-7f, Let-7g, and Let-7i) has been identified in various species, and a number of studies have described the mechanism of Let-7 and its regulation of target genes [17, 18]. Notably, several target genes of Let-7 have been identified in humans (LIN28B, HMGA2, and IGF2BP2) and mice (Igf2bp2, Nras, and Tgfbr1) [19–22]. Although Let-7 is reported to be a tumor suppressor [23, 24], we used anti-Let-7, a modified complementary anti-miR oligomer of Let-7a, to assess the functional efficiency of anti-miR delivery *in vivo*, which is reflected by the expression levels of its target genes.

Here, we suggest an alternative delivery method that could lead to anti-miR accumulation in glioblastoma tumors, and we evaluated its practicality for preclinical study.

## RESULTS

### Evaluation of anti-miR delivery via intratumoral, intrathecal, and intraventricular injection methods

**Figure 1:**
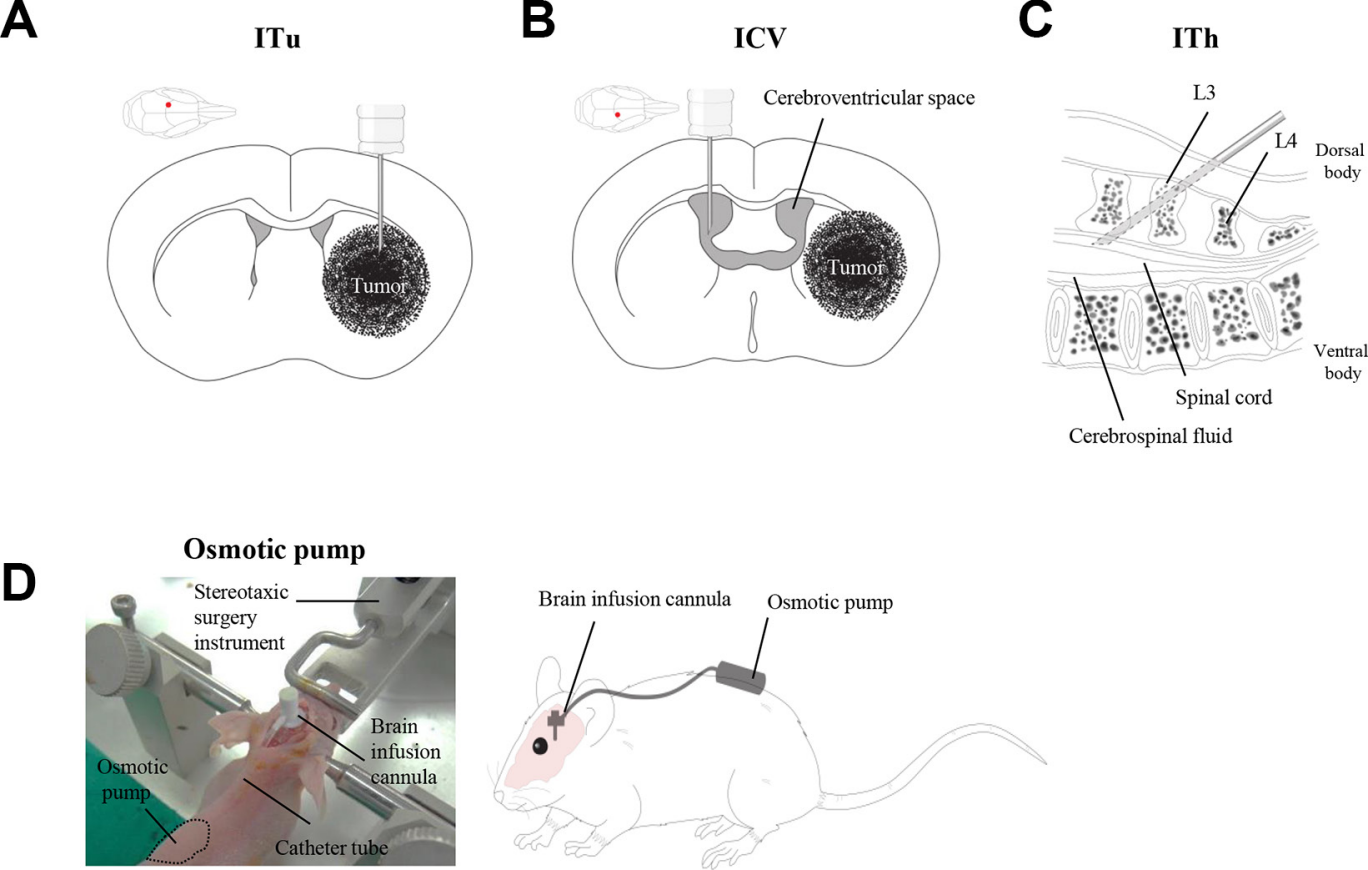
Schematic illustration of the local administration methods Three different routes were employed for the evaluation of anti-miR delivery efficacy: (**A**) Intratumoral injection (ITu). (**B**) Intraventricular injection (ICV). (**C**) Intrathecal injection (ITh). (**D**) Stereotaxic surgery was performed to administer anti-miR into the mouse brain. A brain cannula was implanted with anti-miR-filled osmotic pumps for continuous administration.

We investigated a novel delivery method for anti-miR administration in glioblastoma. To determine the optimal delivery method in glioblastoma preclinical models, three different administration routes were evaluated in this study: intratumoral (ITu), intraventricular (ICV), and intrathecal (ITh). The ITu method can deliver a drug directly to the tumor [25], while both the ICV and ITh methods administer the therapy into the cerebrospinal fluid (CSF) [4]. To examine the efficiency of delivery, we administered anti-Let-7 into the brain of mice via the ITu, ICV, or ITh route by bolus (Figure 1A, 1B and 1C) and osmotic pump (Figure 1B). The expression levels of the target genes of Let-7 were used to determine the efficiency of delivery. The target genes of anti-Let-7 in mice, Igf2bp2, Nras, and Tgfbr1, were evaluated. Let-7 has been shown to play an important role in *Drosophila melanogaster*, and its sequence is well conserved among different species [26]. There have been numerous reports recently implicating Let-7 in cell growth, differentiation, and tumorigenesis [27, 28]. In addition, among the recently reported miRNAs, the target genes of Let-7 have been well validated in different species, including humans (HMGA2, IGF2BP2, and LIN28B) and mice (Igf2bp2, Nras, and Tgfbr1) [29]. Moreover, moderate expression of Let-7 was observed in brain tissues, with only scattered expression in other organs [30]. Although a number of studies have reported that Let7 acts as a tumor suppressor by targeting oncogenic genes, we employed a Let-7-targeting anti-miR to evaluate the distribution pattern after the anti-miR was released into the mouse brain and assessed gene expression levels to predict miRNA function in various regions of the mouse brain.

**Figure 2:**
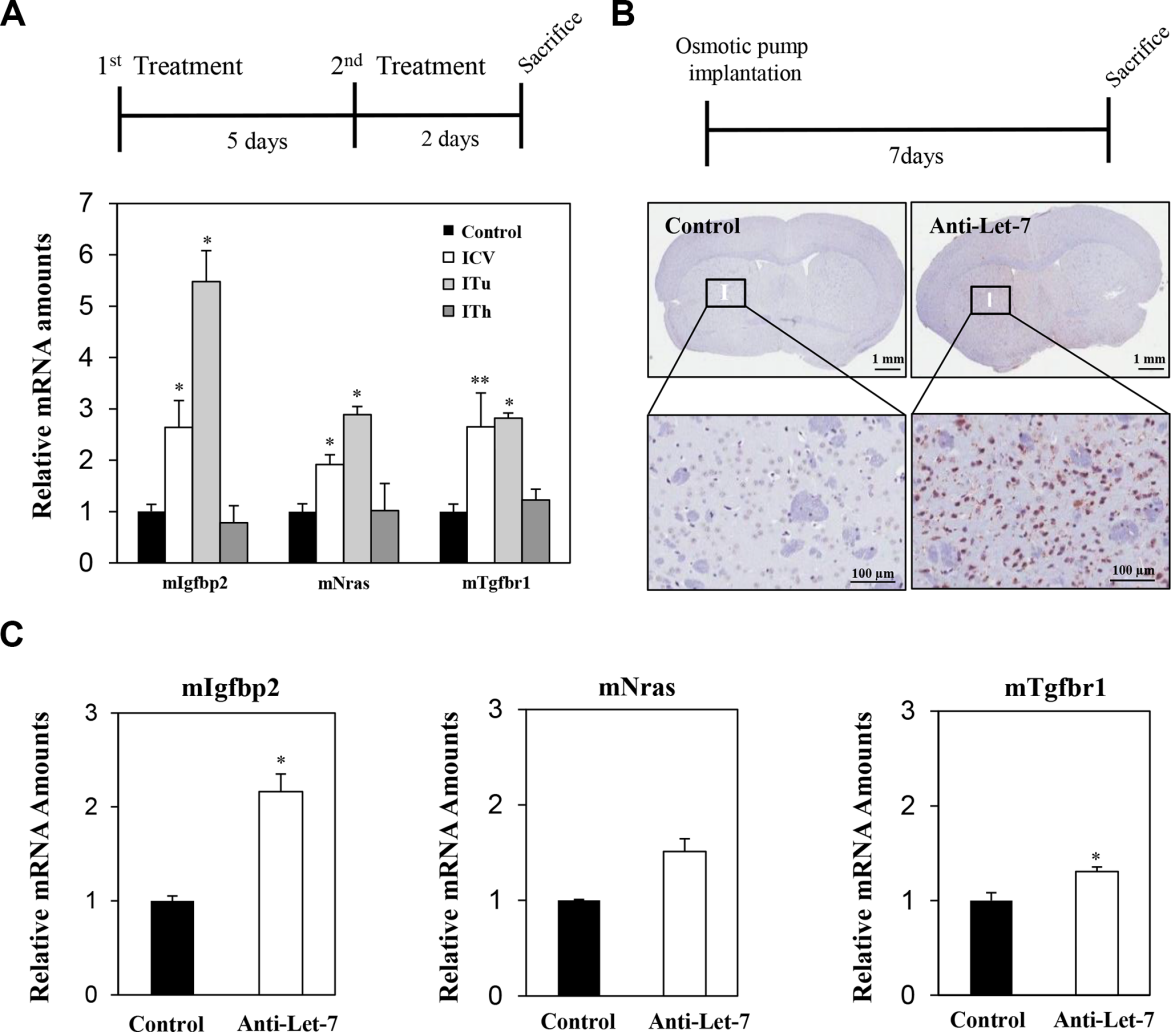
Local delivery of an anti-Let-7 bolus or continuous administration to the brain in a non-tumor-bearing mouse model (**A**) Detailed experimental schedules using non-tumor-bearing mice (BALB/c-nu, 7 weeks) are illustrated (upper). Anti-Let-7 was administered twice (250 μg/5 μL each time, *n* = 10) in the parenchymal (stereotaxic coordinate site of ITu administration, *n* = 10) and lateral cerebroventricular space (stereotaxic coordinate site of ICV administration, *n* = 10) of the mouse brain, and all mice were sacrificed 2 days after the last treatment. qRT-PCR analysis of target gene expression (miRNA-Let-7 targeting mRNAs in mouse tissue: mIgfbp2, mNras, and mTgfbr1) in the mouse brain after applying the different routes of administration (bottom). (**B**) Detailed experimental schedules of osmotic pump surgery in the non-tumor-bearing mice (BALB/c-nu, 7 weeks, *n* = 10 each group) are illustrated (upper). An osmotic pump was used to continuously administer anti-Let-7 (35 μg/day for 7 days). Immunohistochemical analysis using an anti-miR specific antibody in the mouse brain after continuous administration (35 μg/day for 7 days) of anti-Let-7 by osmotic pump (bottom). (**C**) qRT-PCR analysis of Let-7-targeting genes in mouse tissue (mIgf2bp2, mNras, and mTgfbr1) at the injection site (I) for anti-Let-7 (35 μg/day for 7 days) using an osmotic pump. Data are presented as means ± S.E.M. *p < 0.05, **p < 0.01, and *** *p* < 0.001 compared with the control.

As shown in Figure 2A, a significant increase in the expression of Let-7 target genes was observed in the parenchymal tissue of mice injected with anti-Let-7 via the ITu or ICV route, but no significant differences were observed when the mice were injected intrathecally. To investigate whether anti-miR treatment could penetrate the parenchymal region of the mouse brain upon continuous administration, an osmotic pump (35 μg/day) was implanted and maintained for 7 days. We performed immunohistochemical staining using an oligomer-specific antibody and qRT-PCR analysis to assess the distribution of the oligomer in the brain. In the anti-Let-7-treated mouse brain, positively stained cells were observed near the original site of anti-miR injection. However, we did not detect any positive signals at the opposite side of the hemisphere (Figure 2B). Furthermore, the expression levels of anti-Let-7 target genes (Igf2bp2, Nras, and Tgfbr1) were elevated in the group treated with anti-Let-7 compared with the control group (Figure 2C). Both histochemical and mRNA expression analyses showed a correlation, suggesting anti-miRs can be delivered into the mouse brain via ITu and ICV routes, but not the ITh route.

### Differential expression of anti-Let-7 target genes *in vitro*

**Figure 3:**
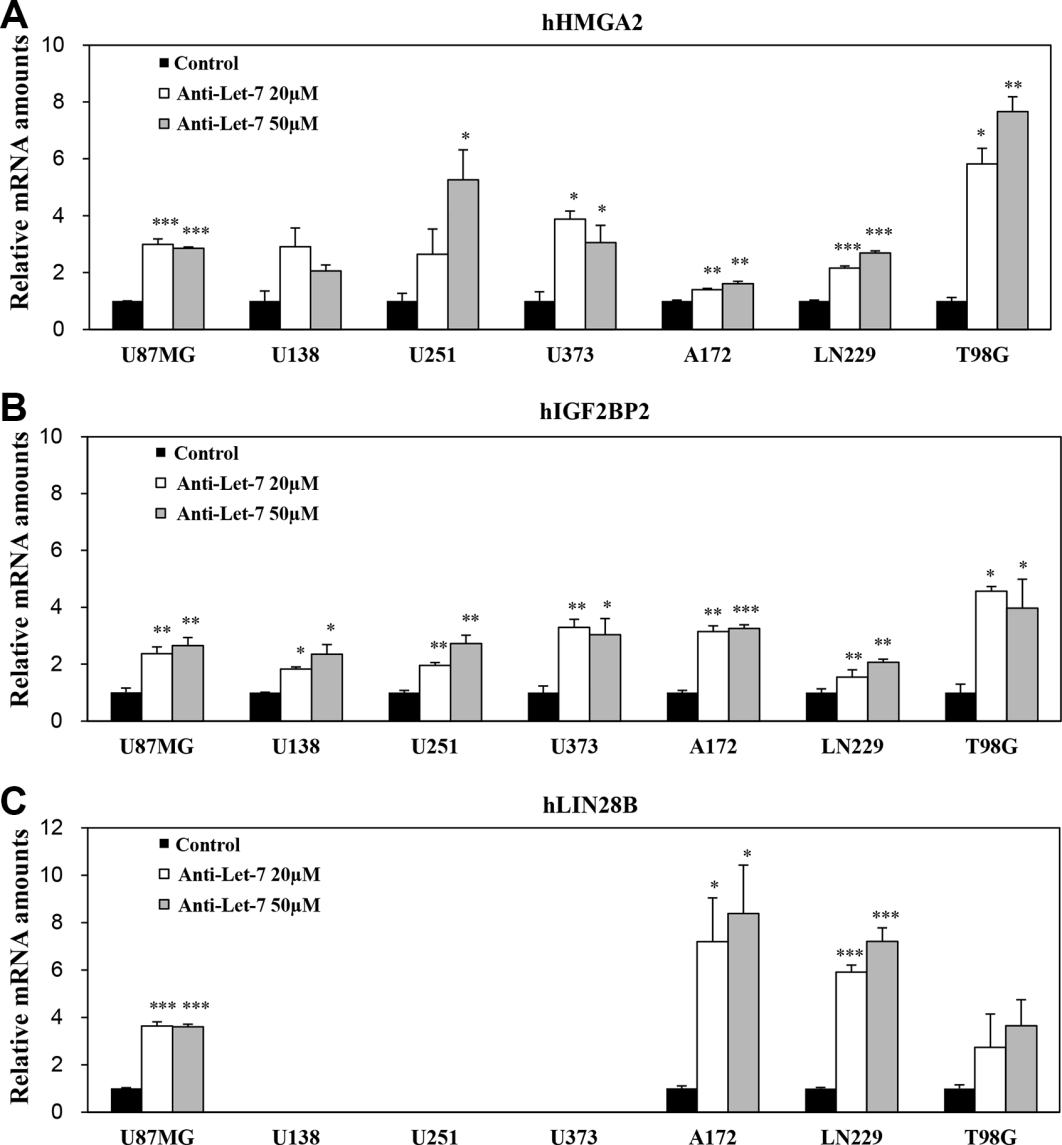
Anti-Let-7 mediates differential expression of its target genes in glioblastoma cell lines and glioblastoma patient-derived cells Quantitative RT-PCR (qRT-PCR) was used to determine the expression of target genes. qRT-PCR analysis of (**A**) hHMGA2, (**B**) hIGF2BP2, and (**C**) hLIN28B mRNA expression in glioblastoma cell lines after anti-Let-7 (20 or 50 nM for 48 h) administration. Data are presented as means ± S.E.M. *p < 0.05, **p < 0.01, and ***p < 0.001 compared with the control.

*In vitro* studies were performed to evaluate the internalization of anti-miR-10b in glioblastoma cells without any support from chemical reagents. We treated glioblastoma cells directly with anti-miRs and observed target gene expression by qRT-PCR. To assess the functionality of Let-7 in glioblastoma cancer cells, we assessed the expression levels of Let-7 target genes in the glioblastoma cell lines U87MG, U138, U251, U373, A172, LN229, and T98G. In particular, we specifically evaluated the Let-7 human target genes HMGA2, IGF2BP2, and LIN28B, which were found to be de-repressed following anti-Let-7 treatment. The expression of HMGA2 and IGF2BP2 increased in seven established glioblastoma cell lines after anti-Let-7 treatment (Figure 3A and 3B). The expression of LIN28B was also elevated after anti-Let-7 treatment, although this effect was not observed in all of the cell lines (U138, U251, and U373; Figure 3C). In addition, our results showed that the Let-7 target genes (HMGA2, IGF2BP2, and LIN28B) were significantly upregulated in glioblastoma patient-derived cells (GBM04T) after anti-Let-7 treatment (Supplementary Figure S1). Recently, various chemical reagents and nanoparticles have been reported for their support in transferring oligomer and chemical drugs. These chemical reagents are useful tools for the delivery of oligomers *in vitro*, although toxicity remains an issue *in vivo*. Our results suggest that this anti-miR, developed by Regulus Therapeutics (San Diego, CA, USA), can be internalized to regulate gene expression in glioblastoma cells without the support of chemical reagents.

### Evaluation of anti-miR delivery to tumor-bearing mice via intratumoral, intrathecal, and intraventricular injection methods

**Figure 4:**
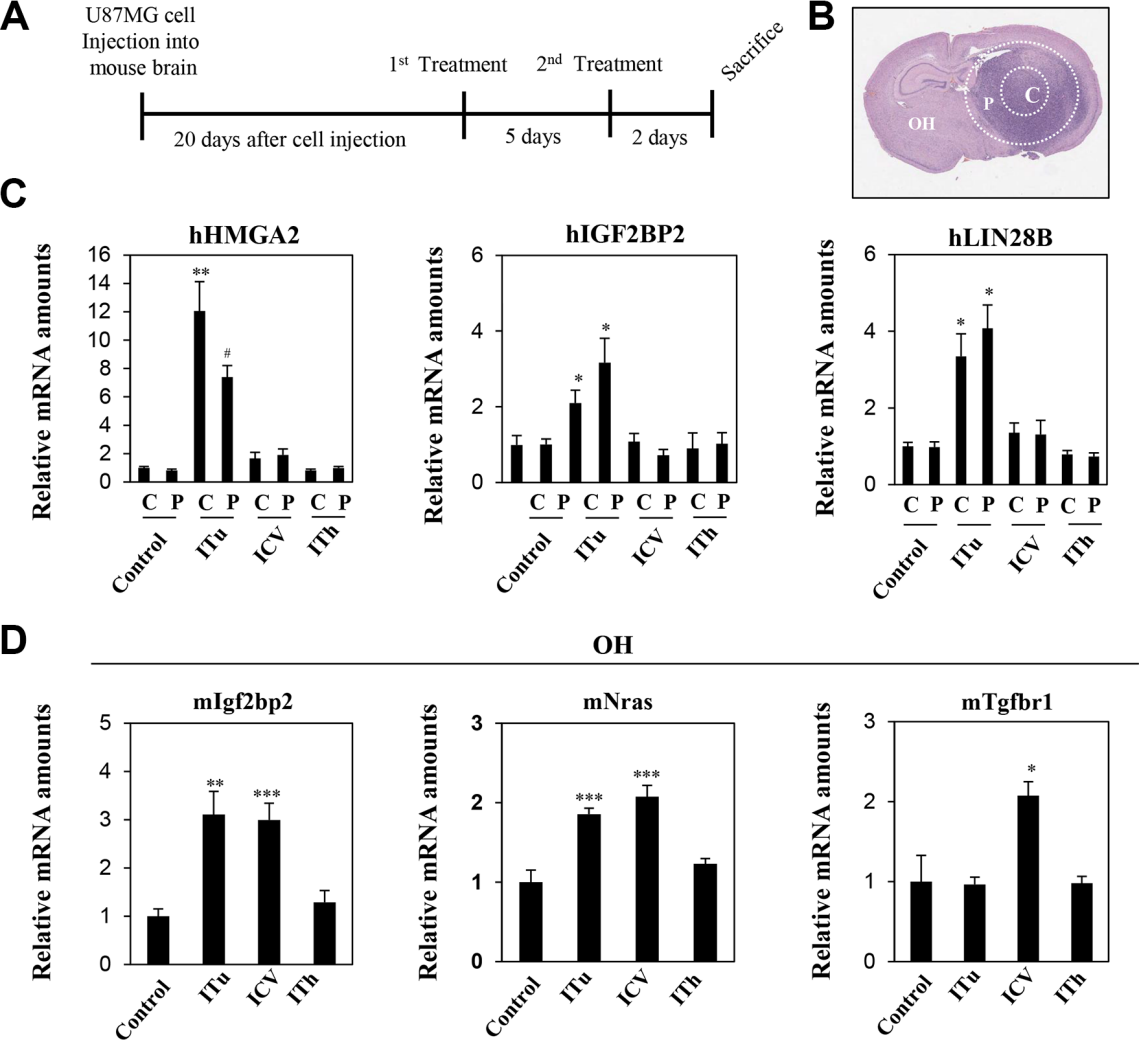
Expression levels of target genes were analyzed to evaluate the efficiency of intratumoral, intraventricular, and intrathecal administration of anti-Let-7 in the U87MG xenograft model (**A**) U87MG cells (2 × 10^5^ Cells/5 μL HBSS, *n* = 10 each group) were injected intracranially using a stereotaxic apparatus in the mouse brain. Twenty days after cell injection, anti-Let-7 was administered twice (250 μg/5 μL each time) via the three different routes of administration. The stereotaxic apparatus was used for the ITu and ICV methods of administration. (**B**) Tissues were isolated from different sites in the mouse brain: tumor core (c), tumor periphery (p) and the opposite hemisphere. (**C**) qRT-PCR analysis of target gene expression (Let-7-targeting mRNAs in human tissue: hHMGA2, hLIN28B, and hIGF2BP2) in the tumor core (c) and periphery (p). (**D**) qRT-PCR analysis of target gene expression (Let-7-targeting mRNAs in mouse tissue: mIgfbp2, mNras, and mTgfbr1) in the opposite hemisphere. C: tumor core, P: tumor periphery, OH: opposite hemisphere. Data are presented as means ± S.E.M. *p < 0.05, **p < 0.01, and ***p < 0.001 compared with the control.

To investigate anti-miR distribution in tumor-bearing mice, U87MG orthotopic xenograft models were employed in this study. The U87MG glioblastoma cell line was implanted using stereotaxic surgery, and anti-Let-7 was administered via bolus injection through three different routes 20 days after the implantation of U87MG cell line (Figure 4A). Brain tissues were isolated and sliced to 2 mm thick. To analyze target gene expression, tissue samples from the tumor core and peripheral regions of the injected hemisphere as well as normal brain tissue on the opposite hemisphere were separated into distinct groups (Figure 4B). The expression levels of anti-Let-7 target genes (in humans: HMGA2, IGF2BP2, and LIN28B; in mice: Igf2bp2, Nras, and Tgfbr1) were examined in each tumor mass and brain tissue from mice.

Three human target genes of anti-Let-7 (HMGA2, LIN28B, and IGFBP2) were upregulated in both the tumor core and peripheral tumor region after ITu injection only (Figure 4C). In the hemisphere opposite to the U87MG-injected site, the expression of Igf2bp2 and Nras were increased by the ICV and ITu routes, while the expression of Tgfbr1 was increased by the ICV method only (Figure 4D). Overall, ITu was the most efficient method for delivering anti-Let-7 to the tumor core and normal peripheral brain tissue, whereas the efficiency of the ICV method was limited despite easy dispersion into normal brain tissue.

### Intratumoral delivery efficacy of anti-miR through an osmotic pump system in glioblastoma xenograft models

**Figure 5:**
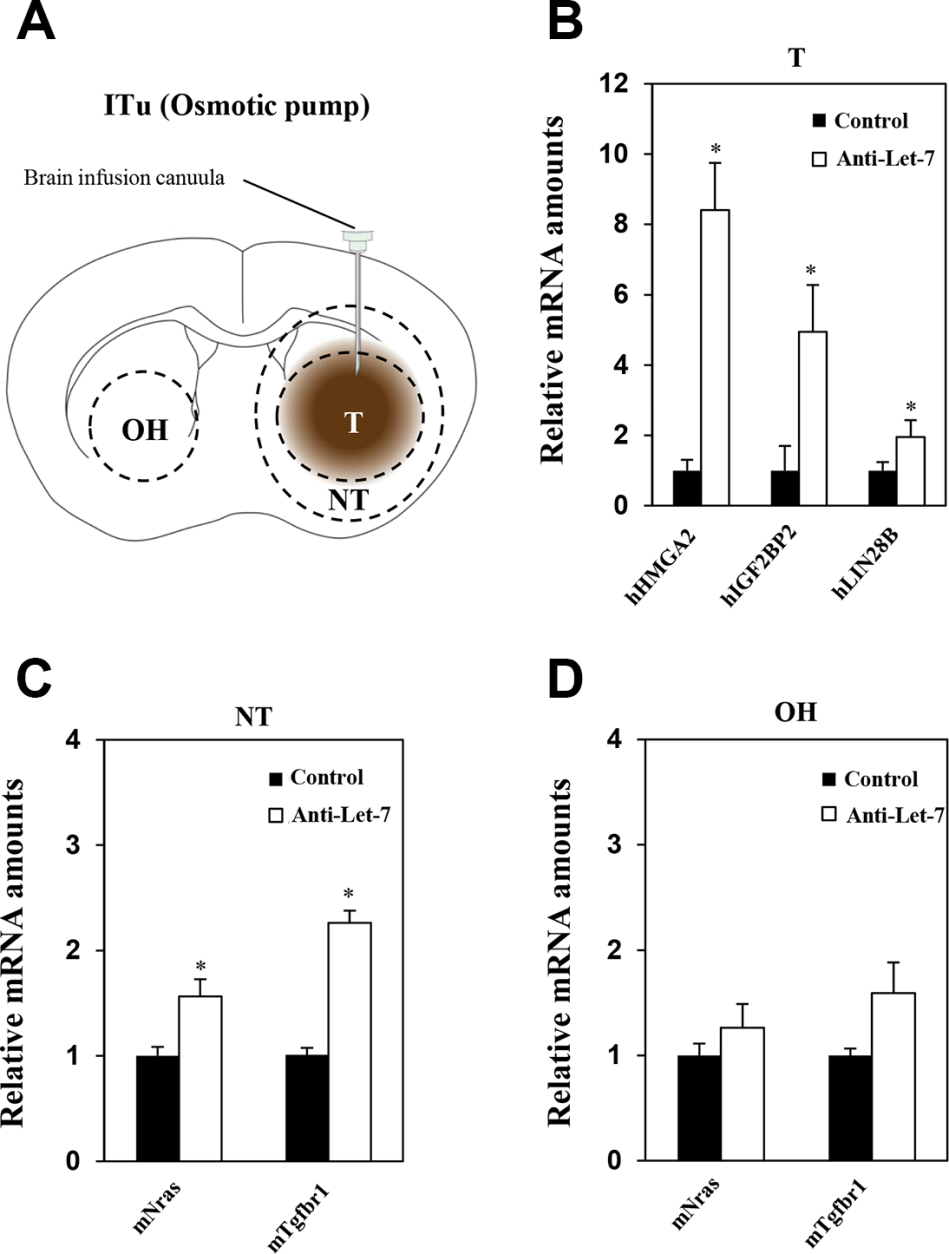
Anti-Let-7 is delivered directly into the brain tumor by intratumoral administration via osmotic pump in U87MG orthotopic and glioblastoma patient-derived xenograft models U87MG cells (2 × 10^5^/5 μL HBSS, *n* = 10 for each group) were implanted into mouse brains using the stereotaxic apparatus. The brain cannula was implanted 20 days after U87MG cell implantation using anti-miR-filled osmotic pumps. (**A**) The osmotic pump was inserted into the mouse subcutaneously, and the cannula was located at the coordinate site of intratumoral administration in the mouse. Anti-Let-7 was released (35 μg/day) into the brain tumor for 7 days via the brain cannula and osmotic pump. (**B**) qRT-PCR analysis of target gene expression (miRNA-Let7-targeting mRNAs in mouse tissue: mIgfbp2, mNras, and mTgfbr1) in brain tumors from U87MG orthotopic models after anti-Let-7 administration. (**C**, **D**) qRT-PCR analysis of Let-7 target gene expression in mouse tissue (mIgfbp2, mNras, and mTgfbr1) in nearby tumor and opposite hemisphere tissue from U87MG orthotopic models after anti-Let-7 administration. T: tumor, NT: normal tissue nearby tumor, OH: opposite hemisphere. Data are presented as means ± S.E.M. *p < 0.05, **p < 0.01, and ***p < 0.001 compared with the control.

For most newly diagnosed glioblastoma patients, the standard therapy protocol is surgical resection of the tumor followed by postoperative radiotherapy with temozolomide treatment [31]. Surgical removal and radiation therapy have resulted in improvement in patient prognosis; however, tumor recurrence occurs in almost all glioblastoma patients after standard therapy. Although surgical removal of the recurring tumor should be considered in all patients, such surgical approach has a number of limitations [32]. Thus, we decided to investigate alternative continuous administration of therapy into the tumor mass in an effort to identify possible new treatment strategies to applicate with surgical resection. To investigate continuous anti-miR administration via the ITu route, anti-Let-7 was administered using an osmotic pump (Figure 5A). Osmotic pumps were filled with an anti-Let-7 solution (*n* = 10, 35 μg/day) or saline (*n* = 10) and connected to the intra-brain cannula to administer the anti-Let-7 at the site of tumor implantation. An intra-brain cannula was implanted into the mouse at brain 20th day after post-implantation U87MG cell, and then anti-Let-7 was delivered continuously for 7 days afterwards. We observed a significant increase in target gene expression in the tumor and adjacent regions compared with the control group given saline (Figure 5B and 5C). Furthermore, the parenchymal tissue from the opposite hemisphere did not exhibit any changes in anti-Let-7 target gene expression (Figure 5D).

The efficiency of delivery and pattern of distribution was evaluated following anti-Let-7 treatment in the mouse brain according to the expression levels of its target genes. However, Let-7 is not a therapeutically targetable miRNA and therefore was only used to evaluate the delivery methods. To evaluate the therapeutic efficacy of anti-miR treatment by ITu administration, we used an anti-miR targeting miR-10b which is a well-studied oncomir in glioblastoma. Basal expression levels of miR-10b were analyzed by qRT-PCR in U87MG cell line as well as seven different glioblastoma patient-derived primary cells, and found elevated expression levels of miR-10b from almost all seven glioblastoma patients compared to the U87MG cell line (Supplementary Figure S2). Among the patient-derived cells, GBM04T patient xenograft model was used to assess the delivery efficacy of anti-miR-10b and it exhibited similar expression levels of miR-10b as the patient-derived glioblastoma cells. Although recent reports have identified valuable target genes of miR-10b, the pathways have not been fully elucidated, limiting our ability to predict miR-10b functions in GBM. Thus, we employed an anti-miR-10b-specific antibody for immunohistochemistry analysis. When anti-miR-10b was administered through ITu route, anti-miR-10b positive cells were more frequently observed in the tumor mass in a dose-dependent manner (Supplementary Figure S3). In addition, when we evaluated the therapeutic efficacy of anti-miR-10b that was released via ITu route in GBM07T glioblastoma patient-derived xenograft model, which exhibited the highest expression of miR-10b among the seven patients, the anti-miR-10b administered group showed a significant improvement in median survival compared to the control group (median survival of control group: 89 days, median survival of anti-miR-10b-treated group: 104 days, *p* = 0.0108; Supplementary Figure S5) Accumulation of Anti-miR-10b was also observed in the tumor mass by immunohistochemistry analysis (Supplementary Figure S4). However, Anti-miR10b did not affect *in vivo* survival in GBM04T patient xenograft model (median survival of control group: 45 days, median survival of anti-miR-10b treated group: 47 days; Supplementary Figure S5). Taken together, these data indicate that the ITu route is a promising route of administration for anti-miR treatment. Furthermore, this approach will be valuable for the development of novel therapeutic options to target highly expressed oncomirs such as miR-10b in glioblastoma.

### Continuous administration of anti-Let-7 by intraventricular delivery in the U87MG orthotopic xenograft model

**Figure 6:**
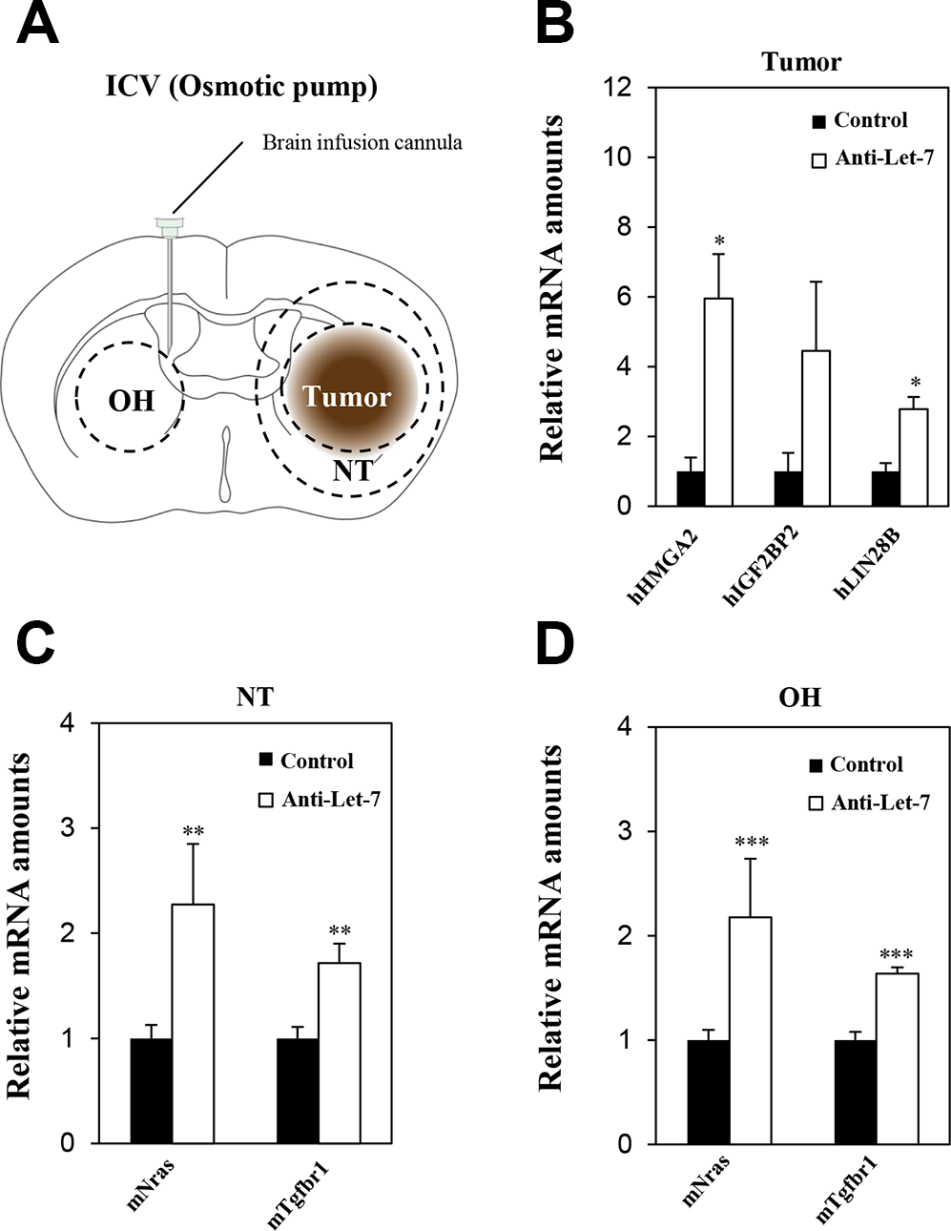
Anti-Let-7 is delivered directly into the cerebrospinal fluid by intraventricular administration via osmotic pump in U87MG orthotopic xenograft models U87MG cells (2 × 10^5^ Cells/5 μL HBSS, *n* = 10 each group) were implanted using the stereotaxic coordinate site in the mouse brain. (**A**) Anti-Let-7 was released (35 μg/day) into the brain for 7 days using an osmotic pump and brain infusion cannula located at the site of intraventricular administration. (**B**) qRT-PCR analysis of target gene expression in the tumor of the U87MG orthotopic model after administration of anti-Let-7. (**C**, **D**) qRT-PCR analysis of target gene expression in normal nearby tumor tissue and the opposite hemisphere of the U87MG orthotopic model after administration of anti-Let-7. T: tumor, NT: normal tissue nearby tumor, OH: opposite hemisphere. Data are presented as means ± S.E.M. *p < 0.05, **p < 0.01, and ***p < 0.001 compared with the control.

Although the delivery of anti-miR by bolus administration via ITu route into the tumor was efficient, there was limited delivery to the entire brain. Therefore, an ICV method combined with an osmotic pump seemed to be a more optimal route for targeting multifocally spread and seeded cancer cells in glioblastoma patients after standard therapy in a clinical environment. Our previous results also showed that bolus administration of anti-Let-7 through the ICV route significantly increased the expression of anti-Let-7 target genes in the opposite hemisphere region. However, no differential expression was found in the tumor core or periphery (Figure 4). Thus, we used the osmotic pump to administer anti-Let-7 continuously into the cerebroventricular space of the mouse brain. Osmotic pumps were filled with an anti-Let-7 solution (*n* = 10, 35 μg/day) or saline (*n* = 10) and placed in a subcutaneous pocket. An intra-brain cannula was connected to the osmotic pump and implanted into the mouse brain 20 days after the U87MG cell implantation. Afterwards, anti-Let-7 was released continuously for 7 days into the ventricular space. Consistently, anti-Let-7 administration using the osmotic pump increased the expression of the human Let-7 target genes, HMGA2, IGF2BP2, and LIN28B (Figure 6A and 6B). In addition, we found that the expression levels of Nras and Tgfbr1 were significantly elevated in both normal tissue adjacent to the tumor and in the opposite hemisphere regions in the oligomer-treated group compared with the saline-administered control (Figure 6C and 6D). Our study suggests that continuous administration using the ICV method successfully delivers anti-miRs to the implanted tumor core as well as to non-neoplastic tissues, which would be an optimal treatment option for treating glioblastoma tumors.

## DISCUSSION

Therapeutic anti-miRs are currently being developed for cancer therapy; however, this is still at an early stage and has not yet been introduced in a clinical setting [14]. Furthermore, applying anti-miR treatment to glioblastoma patients proves to be more challenging. Although multiple anti-cancer drugs are presently available, the majority of them are not suitable for glioblastoma therapy because of the lack of sufficient transportation methods across the BBB following systemic administration. The BBB, comprised of cells exhibiting incredibly strong tight junctions, is a major defense mechanism for the brain, as it helps to prevent outside substances, including microorganisms and chemical agents, from entering the brain. For this reason, the administration of various neurological drugs as well as therapeutic anti-miRs has been unsuccessful. Thus, our study evaluated different administration methods for delivering anti-miRs to the brain and tumor regions, which may be important for further preclinical and clinical studies [33].

Different administration methods are currently being used to deliver drugs directly into the brain, thereby bypassing the BBB [34]. Rather than relying on transporters to pump anti-miRs across the BBB, administration of anti-miRs directly into the CSF or extracellular space of the brain seems to be a more promising therapeutic approach. Among the different delivery methods, the ITh route has been used for administration of drugs into the lumbar subarachnoid space, while the ICV method has been used for drug injection or infusion into the lateral ventricle space of the brain [35]. These ITh and ICV routes of administration are ideal for situations in which the target is within the subarachnoid space or close to the CSF-brain interface. To directly transport anti-miRs into the brain tumor, the ITu method proved to be the most effective delivery route in glioblastoma orthotopic xenograft models. The ITu route is recognized as a potentially useful method that is being widely explored as a means of delivering large agents. In this study, we evaluated the *in vivo* distribution potency of anti-miRs into the brain through different administration routes in the glioblastoma orthotopic model.

Our results suggest that the ITu and ICV administration routes are possible therapeutic options for the delivery of anti-miRs in glioblastoma. To evaluate anti-miR delivery and distribution rates in the brain, we targeted a well-characterized miRNA, Let-7, and validated its target gene expression in glioblastoma xenograft models [36]. We showed that ITu administration of anti-Let-7 led to a significant decrease in the expression of target genes in the tumor core and periphery. Furthermore, anti-miR administration via the ITu route was found to be an excellent delivery option that enabled the anti-miR to diffuse from the tumor core into the peripheral region. Although the ITu route is a promising method that allows evaluation of the therapeutic effects of anti-miR treatment in preclinical models, ITu still poses limited practical use in the clinical environment for glioblastoma patients. Because the current standard therapy for glioblastoma is surgical resection followed by radiotherapy and systemic temozolomide treatment, it will likely be difficult to apply repetitive administration of anti-tumor drugs via the ITu route [37]. In terms of a clinical approach, ICV is more effective for releasing and improving drug delivery into the tumor.

It has been reported that convection-enhanced delivery methods increase the efficacy of anti-cancer drugs in glioblastoma. Convection-enhanced delivery can deliver an anti-cancer agent directly into the brain tumor and the surrounding parenchymal region via continuous release or use of a nanoparticle. Among them, the osmotic pump system is one of the most useful convection-enhanced delivery methods for delivering drugs to brain tumors and bypassing the BBB [38]. Here, we demonstrated that continuous administration of anti-Let-7 by ICV resulted in down-regulation of Let-7 target genes in the tumor as well as in normal brain tissues (Figure 2B, 2C, 4, and 5). Notably, target gene expression levels were significantly increased in the adjacent tumor region and in non-neoplastic tissues in the opposite hemisphere of the mouse brain when anti-Let-7 was administered continuously through the ICV route. In addition, we evaluated anti-miR-10b, an oncogenic miR-10b anti-miR oligomer, in glioblastoma-derived xenograft models [12, 39]. Earlier studies have described oncogenic miRNAs such as miRNA-135b, miRNA-21, miRNA-10b, miRNA-221, and miRNA-196a as novel biomarkers in glioblastoma [40, 41]. Among them, we found miR-10b to be highly expressed in glioblastoma patient-derived cells (data not shown). To validate the delivery methods, anti-miR-10b was used in glioblastoma patient-derived xenograft models. When we administered anti-miR-10b continuously using an osmotic pump to assess its distribution without any support from chemical reagents, anti-miR-10b was observed in the tumor region (Supplementary Figure S3 and S4). In the present study, the osmotic pump was found to be an ideal method for delivering anti-miRs to the brain tumor.

Finally, these findings demonstrate that anti-miRs are able to penetrate the BBB without the use of any viral or lipid carriers, and ICV administration of anti-miRs could lead to whole-brain distribution, including the tumor region. In this report, we describe different methods of direct administration of anti-miRs that are applicable in preclinical models. Moreover, the ICV method of delivery is a promising approach for anti-miR therapy in glioblastoma that warrants further research. We expect that our findings will support future research into novel therapeutic anti-miRs and help establish therapeutic strategies that will eventually improve the current treatments for glioblastoma patients.

## MATERIALS AND METHODS

### Cell culture and anti-Let-7 treatment

U87MG cells, human glioblastoma cell line, were maintained in Eagle's minimal essential medium (Life Technologies, Carlsbad, CA, USA), and U373, A172, LN229, and T98G cell lines were maintained in Dulbecco's modified Eagle's medium (Life Technologies, Carlsbad, CA, USA) containing 10% fetal bovine serum, 100 U/ml penicillin (100 units/mL, Invitrogen, Carlsbad, CA, USA), and streptomycin (100 mg/mL, Invitrogen, Carlsbad, CA, USA). To evaluate intracellular transfer efficiency of anti-miR in glioblastoma cells *in vitro*, each cell line was plated at 1 × 10^5^ cells/2 mL culture medium per well in a 6-well plate. Glioblastoma cell lines were treated with anti-Let-7 (20 μM, 50 μM) without any chemical reagents in the culture medium. 48 h after the treatment, cells were harvested for target gene expression analysis by qRT-PCR.

### Animals

BALB/c-nu mice (female, 6–7 weeks of age) were used for the *in vivo* studies. Animals were obtained from Orient Bio Inc. (Seongnam, Korea) and maintained under specific pathogen-free conditions in facilities approved by the Association for Assessment and Accreditation of Laboratory Animal Care International in accordance with the current regulations and standards of the Laboratory Animal Research Center at the Samsung Biomedical Research institute (Samsung medical center, Seoul, Korea).

### Glioblastoma orthotopic xenograft model

To produce orthotopic glioblastoma models used in this study, U87MG cells (2 × 10^5^/5 μL Hank's Balanced Salt Solution, HBSS) were injected intracranially (coordinates: 0.5 mm anterior, 1.7 mm lateral, 3.2 mm depth from the bregma) into the left brain of mice using a rodent stereotactic device.

### Intratumoral injection

Animals were positioned in a rodent stereotaxic device after anesthetic sedation using Zoletil 50 (50 mg/kg, i.p., Vribac, Carros, France). Anti-miR was administered into the brain using a stereotaxic surgical device (bolus treatment) and/or osmotic pump with a brain catheter (for continuous treatment). For ITu, anti-miRs were released into the left lateral cerebral hemisphere at the following coordinates from the bregma; 0.5 mm anterior (A/P), 1.7 mm lateral, and 3.2 mm depth (Figure 1A).

### Intraventricular and intrathecal injection

For anti-miR release into the CSF, the ICV and ITh routes were used in BALB/c-nu mice. ICV injection was carried out via stereotaxic surgery, and anti-miRs were administered into the right cerebroventricular space (A/P: +0.2 mm; lateral: +1.0 mm; depth: −2.5 mm from the bregma; Figure 1B). ITh injection was performed in the mouse after anesthesia at the L3, L4 intervertebral space. A volume of 5 μL was administered using a 32 G mouse intrathecal catheter (Alzet^®^ brain infusion kit3, Durect Corporation, Cupertino, CA, USA) connected to a 50 μL Hamilton syringe. The animal was lightly restrained to maintain the dorsal recumbent position and for puncture of the dura through spinous processes by the catheter. Anti-miRs were released into the mouse epidural space, and animals were sacrificed after the final treatment to analyze their distribution in the brain. The detailed course of events is described in Figure 1C.

### Osmotic pump implantation

The mice were anesthetized and immobilized in a stereotactic head frame. Pumps were assembled for intracranial infusion according to the manufacturer's instructions, and both an infusion cannula (1.5 cm tubing) and a 25 gauge 3 mm long needle were attached. A brain cannula was implanted into the left hemisphere, with respect to the bregma/midline intersection (A/P: +0.5 mm; lateral: +1.7 mm; depth: −3.2 mm), for intratumoral injection. For intraventricular injection into the right hemisphere, the cannula was placed at the following coordinates from the bregma: +0.2 mm anterior; −1.0 mm lateral; −2.5 mm depth. An ALZET 1004D (11 μL/h flow rate, 100 μL fill volume, Durect Corporation, Cupertino, CA, USA) osmotic pump filled with anti-miR solution was implanted subcutaneously into the mouse along the left side of its back (Figure 1D). After the infusion was complete, mice were anesthetized, and all components of the pump systems were removed to avoid any interference between the pump and anti-miR oligomer.

### Target gene expression levels: qRT-PCR

To analyze the target gene expression levels in mouse brain according to the different treatment routes, brains were isolated after sacrifice and sliced into 2 mm coronal sections using a mouse brain matrix. The brain slices were embedded in a frozen mold using the optimal cutting temperature compound, and tissue samples were obtained from the tumor, tumor periphery, and the opposite hemisphere using a microsurgical blade after selective co-ordinate sites in the mouse brain tissues were determined by hematoxylin and eosin staining. Tissue samples and *in vitro* cultured cells were frozen in liquid nitrogen, and total RNA was isolated using the RNeasy Plus Mini Kit (Qiagen, Valencia, CA, USA), and equal amounts of RNA were subjected to cDNA synthesis using the SuperScript^™^ III First-strand cDNA Synthesis Kit (Life Technologies, Carlsbad, CA, USA). The expression levels of human mRNAs (HMGA2: HS00171569_M1, hIGF2BP2:HS00198023_M1, LIN28b: HS01013729_M1, and GAPDH: HS02758991_G1) were quantified using TaqMan assays (Life Technologies, Carlsbad, CA, USA), and the expression levels of mouse mRNAs (Igf2bp2 primers: forward 5′-TGT TGG ATG GGC TGT TGG-3′; reverse 5′-TCA AAC TGA TGC CCA CTG AG-3′, Nras primers: forward 5′-CGA TCC AGC TAA TCC AGA ACC-3′; reverse 5′-CTC TCA TGG CAC TGT ACT CC-3′, Tgfbr1 primers: forward 5′- CCA AAC CAC AGA GTA GGC AC-3′; reverse 5′-ACC AAT AGA ACA GCG TCG AG-3′, Gapdh br1 primers: forward 5′- GTG GAG TCA TAC TGG AAC ATG TAG-3′; reverse 5′-AAT GGT GAA GGT CGG TGT G-3′) were quantified using SYBR green fluorescent dye (Solis Biodyne, Tartu, Estonia). The relative mRNA levels were evaluated using the Applied Biosystems 7900HT Real-Time PCR System (Applied Biosystems, Carlsbad, CA, USA) and normalized to the glyceraldehyde 3-phosphate dehydrogenase level (GAPDH).

### Immunohistochemistry

To analyze the distribution in the mouse brain *in vivo*, we isolated the brain after animal sacrifice. Serial 4 μm thick paraffin sections were subjected to immunohistochemical staining. To detect the anti-miR, the sections were stained with a rabbit anti-oligomer specific antibody obtained from Regulus Therapeutics (San Diego, CA, USA).

### Design of the anti-miR oligomer

The anti-miR oligomer was a kind gift from Regulus Therapeutics. The anti-Let-7 oligomer sequence is 5′-ATATACAACCTACUACCUCA-3′.

### Statistics

The data are presented as means ± S.E.M. Significant differences were analyzed by one-way ANOVA using the SPSS 18.0 program (SPSS Inc, Chicago, IL, USA). Differences were considered significant at *p* < 0.05.

## SUPPLEMENTARY MATERIALS FIGURES


