# Supplementary material for: Anti-miR delivery strategies to bypass the blood-brain barrier in glioblastoma therapy
Source: Oncotarget. 2016 Apr 19;7(20):29400–11. doi: 10.18632/oncotarget.8837 (PMC5045404; doi:10.18632/oncotarget.8837)
Supplement: Supplementary file 1 [file oncotarget-07-29400-s001.pdf]

Anti-miR delivery strategies to bypass the blood-brain barrier in glioblastoma therapy

Supplementary Materials

MATERIALS AND METHODS

Glioblastoma patient-derived cells

In accordance with the patient's informed consent and appropriate review by the IRB (Institutional Review Board), glioblastoma patient-derived cells were obtained from a patient undergoing surgery at the Samsung Medical Center (Seoul, Korea). The tumor sample was classified as glioblastoma based on the pathologist's examination and according to the World Health Organization criteria. Within 1 h following surgical resection, the tumor was mechanically and enzymatically dissociated into single cells. Before injection of the single cells into mice, glioblastoma patient-derived cells were briefly maintained in NBE (Neuronal Basal Medium) neurosphere culture medium consisting of Neurobasal-A medium (Invitrogen, Carlsbad, CA, USA) supplemented with glutamine (2 mM, Invitrogen, Carlsbad, CA, USA), penicillin (100 units/mL, Invitrogen), streptomycin (100 mg/mL, Invitrogen, Carlsbad, CA, USA), recombinant human basic fibroblast and epidermal growth factors (100 ng/mL each, R&D Systems, Minneapolis, MN, USA), and N2 and B27 supplements (0.5 × each, Invitrogen, Carlsbad, CA, USA).

Design of the anti-miR oligomer: Anti-miR-10b

Anti-miR oligomer was a kind gift from Regulus Therapeutics. The anti-Let-10b oligomer sequence is 5′-AATTCGGTUCTACAGGGTA-3′.

Anti-miR treatment *in vitro*

To evaluate intracellular transfer efficiency of anti-miR in glioblastoma patient derived cell *in vitro*, GBM04T cell was plated at 1×10^5 cells/2 mL culture medium per well in a 6-well plate. GBM04T cell was treated with anti-miR-10b (0–100 µM) without any chemical reagents in the culture medium. 48 h after the treatment, anti-miR-10b treated cell was harvested for target gene expression analysis by qRT-PCR.

miR-10b expression analysis by qRT-PCR

Total RNA was extracted using QIAzol lysis reagent (Cat. No. 79306, QIAGEN, Valencia, CA, USA) and purified with miRNeasy mini kit (cat No. 217004, QIAGEN, Valencia, CA, USA). miRNA specific qRT-PCR by Tagman Array miRNA were used for quantification of miR-10b expression. microRNA specific cDNA was synthesized using Tagman MicroRNA Reverse Transcription kit (Cat. No. 4366596, Applied Biosystems, Carlsbad, CA, USA). and reverse transcriptase reaction products were added to 20 × Tagman Universal PCR Master Mix to final volume of 5 µL per reaction samples. For miRNA analysis, miR-10b Tagman probe (Has-miR-10b, Cat. No. 4427975, Life Technologies, Carlsbad, CA, USA) was purchased and PCR was run on CFX ConnectTM Real-Time PCR (Bio-Rad, Hercules, CA, USA).

Immunohistochemistry quantification

To analyze anti-miR-10b distribution in the mouse brain *in vivo*, we produced a serial of 4 µm thick paraffin sections and were subjected for immunohistochemical staining after anti-miR-10b administration. For immunohistochemistry, primary antibody against anti-miR (supplied form Regulus therapeutics, 1:1000) was used and the resultant sections were pathologically confirmed for tumor phenotype and specific immunostaining. The positive cells were counted and analyzed by HistoQuest 3.0 software (Tissue Gnostics, Vienna, Austria).

Statistics

To analyze statistical comparisons of tumor regression and the significance of differences in overall survival. we measured the Mantel– Cox log-rank test and Kaplan-Meier graph represented using Prism5 software. All data are presented as mean ± S.E.M and significance level of $p < 0.05$ was used for all tests.

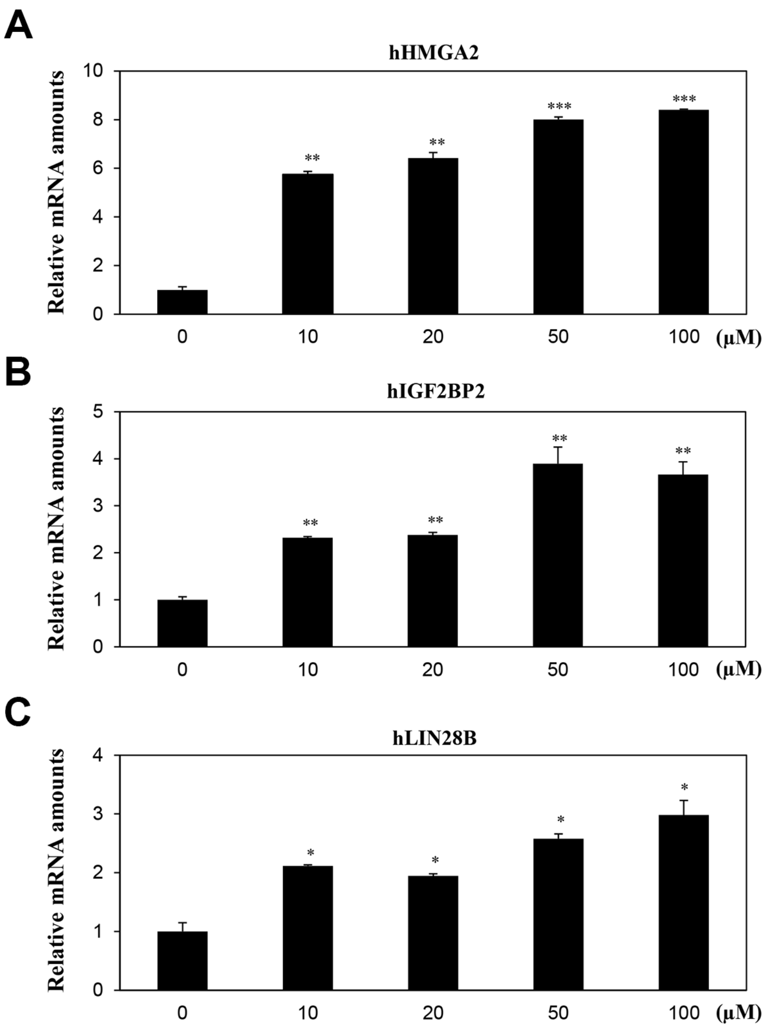

Supplementary Figure S1: Antisense oligomers mediate differential expression of target genes in glioblastoma patient-derived cell. (**A**) qRT-PCR analysis of hHMGA2 mRNA expression in anti-Let7-treated (10–100 μM, for 48 h) glioblastoma patient-derived cells (GBM04T). (**B**) qRT-PCR analysis of hIGFBP2 mRNA expression in anti-Let7-treated (10–100 μM, for 48 h) GBM04T cells. (**C**) qRT-PCR analysis of hLIN28B mRNA expression in anti-Let7-treated (10–100 μM, for 48 h) GBM04T cells. Data are presented as means ± S.E.M. *$p < 0.05$, **$p < 0.01$, and ***$p < 0.001$ compared with the control.

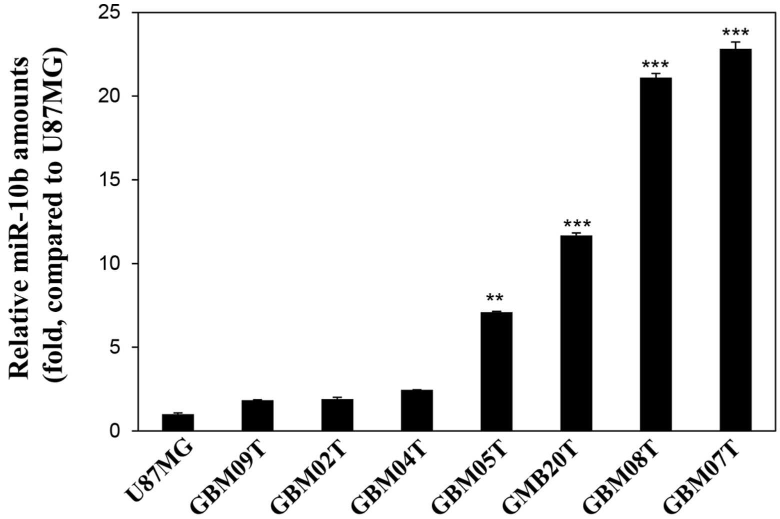

Supplementary Figure S2: Basal expression levels of miR-10b in U87MG glioblastoma cell line and glioblastoma patient-derived cells. miR-10b expression levels were evaluated by qRT-PCR in U87MG and glioblastoma patient-derived cells (seven patients). Relative miR-10b expression levels were calculated according to the ΔCT values. Data are presented as means ± S.E.M. ***p* < 0.01 and ****p* < 0.001 compared with U87MG cells.

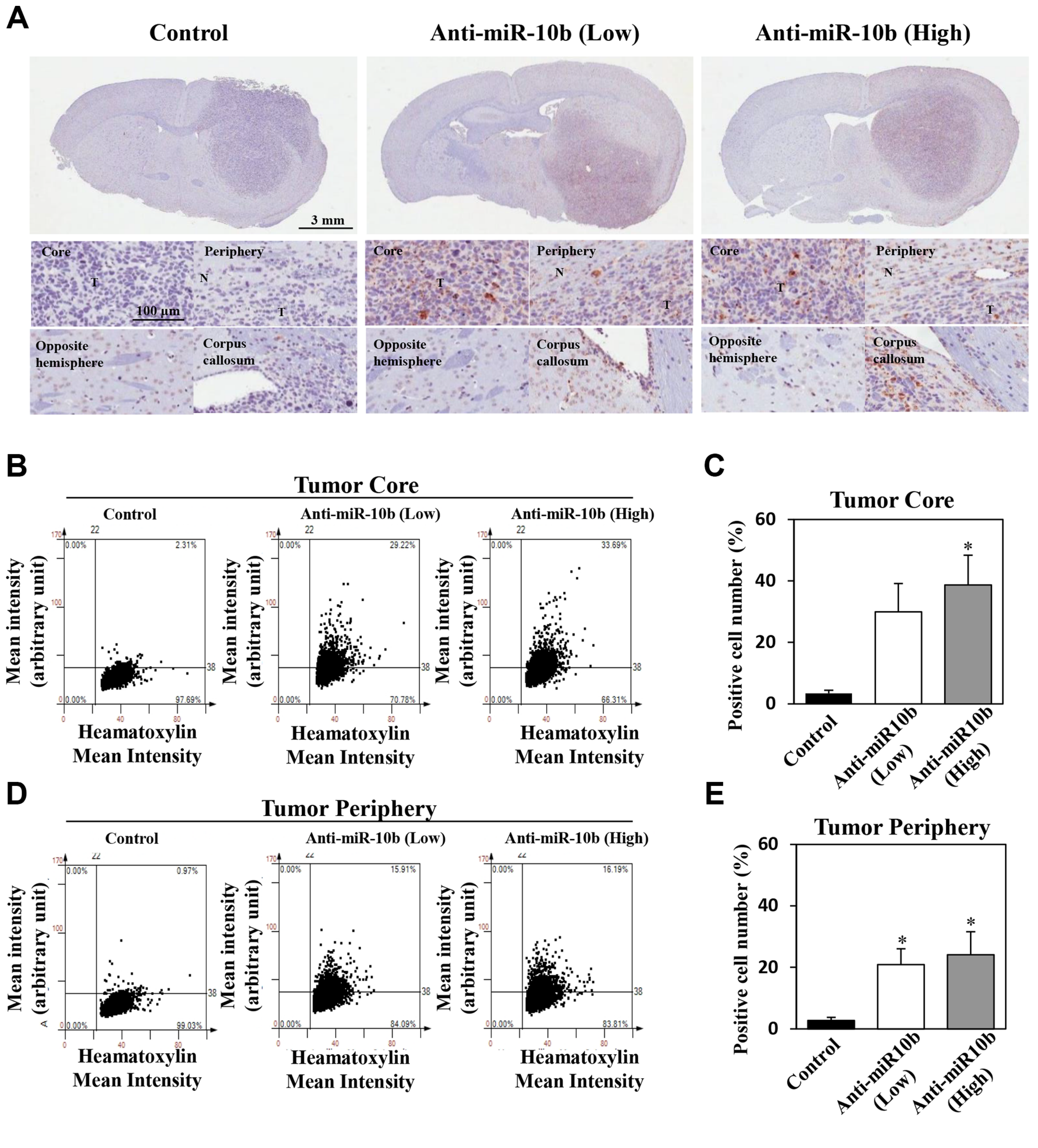

Supplementary Figure S3: Anti-Let10b is delivered directly into cerebrospinal fluid by intratumoral administration via an osmotic pump in the glioblastoma patient-derived xenograft model. GBM04T glioblastoma patient-derived cells (2×10^5/5 µL HBSS) were implanted in the stereotaxic coordinate site in the mouse brain. An osmotic pump was inserted in the mouse subcutaneously, and the cannula was placed at the site of intratumoral administration in the mouse 20 days after cell implantation. Anti-Let10b was released (Low: 17.5 µg/day, High; 35 µg/day) into the brain tumor for 7 days through the brain cannula and osmotic pump. (**A**) Immunohistochemical analyses using an antisense oligonucleotide specific antibody in the mouse brain. Positively stained cells were observed in mouse brains administered a low (middle) and high dose (right) of anti-miR10b compared with the control group (left). (**B**, **C**) Quantification of representative images show a positive cell population in the tumor core. (**D**, **E**) Quantification of representative images of the tumor periphery. The images were analyzed using the HistoQuest software. Data are presented as means ± S.E.M. *$p < 0.05$, **$p < 0.01$, and ***$p < 0.001$ compared with the control.

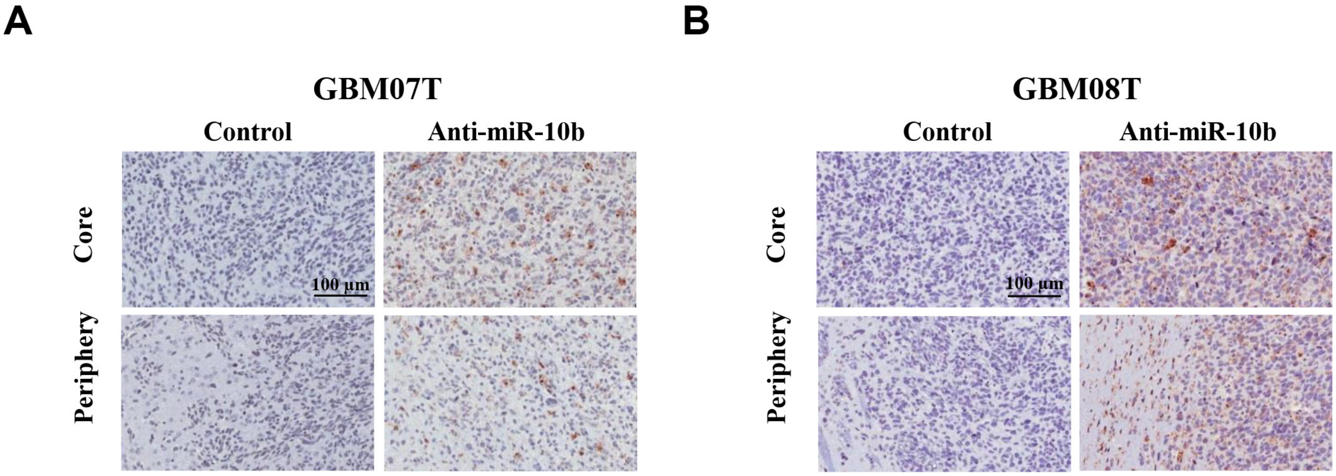

Supplementary Figure S4: Anti-Let10b is delivered directly into the cerebrospinal fluid by intraventricular administration via osmotic pump in the glioblastoma patient-derived xenograft model. Glioblastoma patient cells with high expression of miR10b (GBM07T and GBM08T) were implanted in the mouse brain at the stereotaxic coordinate site. An osmotic pump was inserted in the mouse subcutaneously, and the cannula was placed at the coordinate site of intratumoral administration in the mouse 40 days after cell implantation. Anti-Let10b was released (35 µg/day) into the brain for 7 days by an osmotic pump and brain infusion cannula located at the site of intraventricular administration. Immunohistochemical analysis using an antisense oligonucleotide specific antibody in the mouse brain of (**A**) GBM07T and (**B**) GBM08T GBM patient xenograft models.

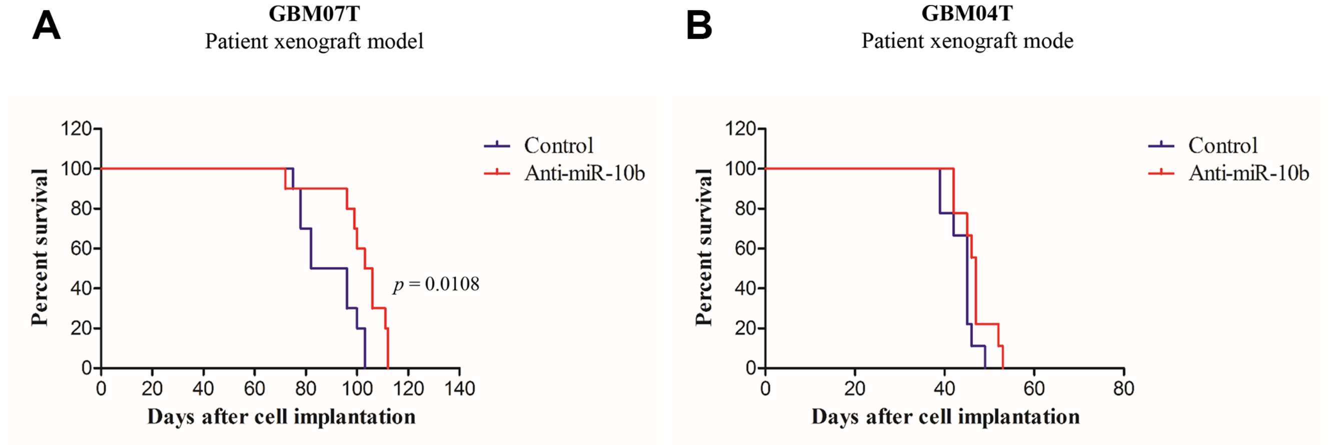

Supplementary Figure S5: *In vivo* survival analysis of glioblastoma patient-derived xenograft models. Glioblastoma patient derived cells (2×10^5/5 µL HBSS, n = 10 each group) were implanted into the mouse brain using the stereotaxic coordinate. An osmotic pump was inserted in the mouse subcutaneously, and the cannula was placed at the coordinate site of intratumoral administration in the mouse brain at half-median survival day (GBM07T: 40th day, GBM04T:20th day) after cell implantation. Anti-miR-10b was released (35 µg/day) into the brain for 7 days by osmotic pump, and the infusion cannula has been placed at the site of intratumoral administration. Therapeutic efficacy was evaluated in (**A**) GBM07T and (**B**) GBM04T glioblastoma patient-derived xenograft models.
